# Computational Pressure-Fluid Dynamics Applied to Index of Microcirculatory Resistance, Predicting the Prognosis of Drug-Coated Balloons Compared With Drug-Eluting Stents in STEMI Patients

**DOI:** 10.3389/fphys.2022.898659

**Published:** 2022-05-24

**Authors:** Yang Duan, Yiwen Wang, Min Zhang, Zhi Li, Lei Chen, Hao Miao, Siyu Pei, Yuan Lu, Zhirong Wang

**Affiliations:** Department of Cardiology, The Affiliated Hospital of Xuzhou Medical University, Xuzhou, China

**Keywords:** computational pressure-fluid dynamics derived index of microcirculatory resistance, drug-coated balloon, ST-segment elevation myocardial infarction, primary percutaneous coronary intervention, retrospective study, major adverse cardiovascular event

## Abstract

**Background:** The impairment of microvascular injury on prognosis has increasingly drawn extensive awareness along with the high morbidity and mortality of ST-segment elevation myocardial infarction (STEMI) over recent years. The prognostic significance of computational pressure-fluid dynamics applied to index of microcirculatory resistance, derived from coronary angiography (CPFD-caIMR) in microvascular injury evaluation of STEMI patients remained inconclusive.

**Methods:** A total of 213 patients who met the inclusion criteria were selected retrospectively from 1003 STEMI patients from February 2018 to February 2020. Propensity score matching (PSM) was thereafter finished. CPFD-caIMR of all patients was obtained off-line using the software (FlashAngio, Rainmed Ltd., Suzhou, China) after PPCI. The primary endpoint was to compare the CPFD-caIMR and the incidence of major adverse cardiovascular events (MACEs) between drug-coated balloons (DCB) and drug-eluting stents (DES) groups. The correlation between CPFD-caIMR and MACEs was analyzed, and the prognosis of patients with STEMI was evaluated by CPFD-caIMR by multivariate regression analysis.

**Results:** Totally 213 STEMI patients with successful primary percutaneous coronary intervention (PPCI) were included, of whom 84 patients accepted DCB and 129 patients accepted DES respectively. Baseline characteristics and CPFD-caIMR were comparable between DCB and DES groups after PSM (62 patients in each group). CPFD-caIMR was not significantly different between two groups (DES vs. DCB: mean difference: 2.26, 95% CI -4.05 to 8.57, *p* = 0.45), and so was it when re-grouped by whether CPFD-caIMR > 40U or not (DES vs. DCB: 34.17% vs. 27.16%, *p* = 0.29). After a follow-up of 1 year, more MACEs occurred in DES group than DCB group (relative risk: 2.50, 95% CI 1.04 to 6.02, *p* = 0.04). The predictors of MACEs by multi-variate analysis found that, only time from symptom to balloon (*p* = 0.03) and time from door to balloon (*p* < 0.01) were independent predictors of MACEs, independent of treatment with DCB or DES intervention. Furthermore, CPFD-caIMR > 40U became an independent predictor of the combined events including cardiovascular deaths or heart failure readmission irrespective of PSM (odds ratio: 4.07, 95% CI: 1.06 to 7.66, *p* = 0.04).

**Conclusion:** CPFD-caIMR was a promising method for prognosis, which can predict CV death or heart failure readmission in STEMI patients. DCB was a possible strategy in PPCI of STEMI patients, not inferior to DES based on microvascular injury evaluated by CPFD-caIMR.

## Introduction

Microvascular injury is closely related to the prognosis of ST-segment elevation myocardial infarction (STEMI) (Alekseeva et al., 2021). Although the blood flow of criminal vessels is restored to Thrombolysis in myocardial infarction (TIMI) Level 3 after primary percutaneous coronary intervention (PPCI), the myocardium perfusion may not effectively resume with existence of coronary microvascular injury (Jaski et al., 2016). Index of microcirculatory resistance (IMR) was reported as a good index to reflect coronary microcirculation and predict the prognosis of STEMI (Cuculi et al., 2014; Carrick et al., 2016). But it was still perceived as a research tool and its application within clinical practice remains extremely limited due to a complex guide wire measurement, at the state of maximum hyperemia taking the risk of hypotension and arrhythmia (Geng et al., 2022). Computational pressure-fluid dynamics derived index of microcirculatory resistance, applied to coronary angiography (CAG, CPFD-caIMR), without extra steps, was proved to have high correlation and diagnostic accuracy with invasive IMR measured by traditional guidewire (Choi et al., 2021). A non-invasive method utilizing computational fluid dynamics to derive caIMR presented a high accuracy (Abuouf et al., 2021). Nowadays there has been rare studies elucidating the effectiveness of drug-coated balloon (DCB) treatment on the prognosis of STEMI patients with PPCI based on microvascular injury evaluation by CPFD-caIMR.

The occurrence of in-stent restenosis (ISR), late stent thrombosis and bleeding caused by long-term dual antiplatelet therapy (DAPT) gave rise to the new concept of “intervention without implantation” which was first emerging in Europe (De Luca et al., 2008; Neumann et al., 2019; Her et al., 2021). DCB can release effective therapeutic concentration of drugs at the lesion site for a short adherent time, thereby reducing restenosis without leaving foreign objects in the blood vessel (Aboyans et al., 2018). DCB was thereafter recommended as a strategy for coronary artery and peripheral vessels diseases. (Neumann et al., 2019; Her et al., 2021). However, DCB has not been recommended as an alternative of drug-eluting stent (DES) in the high-risk conditions like STEMI (De Luca et al., 2008), (Vos et al., 2014; Ho et al., 2015; Nijhoff et al., 2015; Gobić et al., 2017). From already published randomized controlled trials (RCTs) performed in STEMI patients, DCB presented no significant difference of major adverse cardiovascular events (MACEs) and late lumen loss versus DES with a follow-up of both 1 year and 2 years in the REVELATION trial (Vos et al., 2019; Niehe et al., 2022). Similar results were also manifested from another study with a follow-up of 6 mon (Gobić et al., 2017). The above-mentioned results suggested the potential effectiveness of DCB on the prognosis of STEMI patients, which was not inferior to DES in STEMI.

In this study, we intended to assess the effects of DCB on short and long-term prognosis in STEMI patients receiving PPCI by measuring CPFD-caIMR after PPCI, to assess the relationship between CPFD-caIMR and MACEs, and to determine the predictors of MACEs.

## Materials and Methods

### Study Design

This study is a retrospective controlled cohort study. 1003 STEMI patients who underwent PPCI in the Affiliated Hospital of Xuzhou Medical University of China from February 2018 to February 2020 were selected. After strict screening, a total of 213 patients were finally included. Among them, 129 patients were treated by DES and 84 patients were treated by DCB. After 1:1 propensity score matching (PSM), 62 patients from each group who matched baseline characteristics were re-analyzed.

### Study Population and Eligibility

Patients were included if 1) STEMI was diagnosed according to current guidelines (Ibanez et al., 2018); 2) Spontaneous reperfusion of the infarct-related artery (IRA) confirmed by CAG or low-burden thrombus after percutaneous transluminal coronary angioplasty (PTCA) and patients accepted successful PPCI; 3) with complete and available follow-up more than a year; 4) the quality of angiography images met requirements of reconstruction.

Patients were excluded if they are combined with: 1) history of PCI or coronary artery bypass grafting; 2) severe liver and renal dysfunction; 3) occlusive lesions or severe distorted calcified lesions; 4) iodine contrast agent allergy or contraindication of adenosine drugs; 5) severe coagulation dysfunction or hemorrhagic diseases; 6) target lesions involving myocardial bridge; 7) lesions located within 3 mm (excluding 3 mm) at the ostial of the ascending aorta.

### Study Procedures

#### PPCI Procedure

The medical treatments were performed by the same team, according to updated guidelines of STEMI (Ibanez et al., 2018). All patients administered the loading doses of DAPT before the surgery and received PPCI within 90 min after first medical contact (Neumann et al., 2019). Trans-radial or femoral artery was punctured and catheterized to conduct coronary angiography. During the procedure, IRA was merely intervened, using a 70–100 u/kg dosage of unfractionated heparin. GPIIB/IIIA receptor antagonists depended on the individual situation. Patients were selected if lesions had spontaneous reperfusion of the IRA or low-burden thrombus after PTCA confirmed by CAG. And all patients gave the written informed consent in the PPCI process.

The procedure of DCB group: After adequate pre-dilation, 200 ug nitroglycerin was injected to the IRA until the blood flow was restored to TIMI grade 3, with less than 20% residual stenosis and without dissection or dissection below type B. The size of DCB was determined by the diameter of the normal segment of IRA The ratio of the DCB diameter to the artery diameter was 1.0-1.1:1, and the length of DCB should be 3–5 mm beyond the target lesion, with dilation lasting from 40 to 60 s. Considering the comparability of follow-up, patients using Sequent Please DCBs were only selected in this study (B. Braun, Melsungen, Germany). Success criteria: the blood flow of IRA reached TIMI grade 3, residual stenosis less than 20%.

The procedure of DES group: After catheterization, the lesion was first treated by pre-dilated balloon with a low pressure (8–12 atm), using the double guide wire or cutting balloon for adequate dilation if necessary. Then 200 ug nitroglycerin was injected to the IRA and angiography was repeated. The size of DES accorded with the diameter of the IRA. The ratio of stent diameter to target artery diameter was 1.1-1.2:1.0. In case of stent malapposition observed from repeated angiography, the non-compliant balloon would be used for post-dilation. The second-generation DES was used and no significant difference both in ischemia and bleeding risk was observed before between different kinds of DES (Schapiro-Dufour et al., 2019).

After successful surgery, patients were transferred to coronary care unit (CCU) after surgery for continuously intensive drug therapy.

#### CPFD-CaIMR Examination Procedure

**FIGURE 1:**
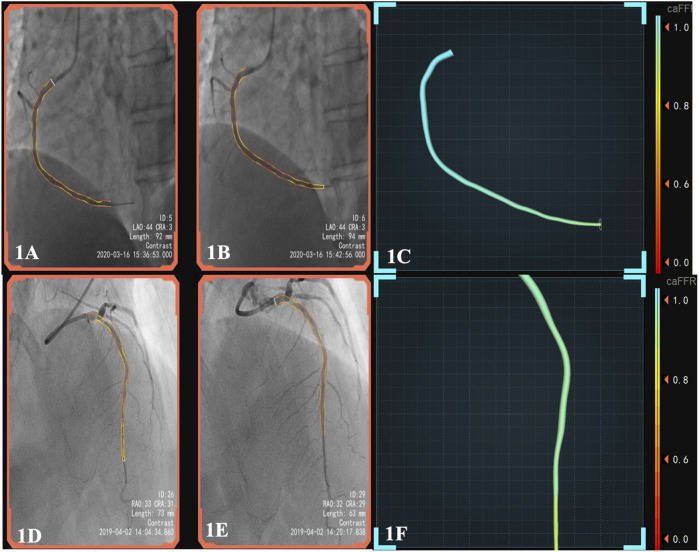
**(A)**, pre-surgery angiography; **(B)**, post-surgery of DCB; **(C)**, the patient’s post-operative caFFR was normal and caIMR was high (caFFR = 0.92, caIMR = 42.1). After 6 months of follow-up, the patient had myocardial infarction again.; **(D)**, pre-surgery angiography; **(E)**, post-surgery of DES; **(F)**, the patient’s postoperative caFFR and caIMR (caFFR = 0.91, caIMR = 22.8) were normal and no MACEs events occurred in 1.5 years of follow-up. Abbreviations: caFFR, coronary angiography-derived fractional flow reserve; caIMR, coronary-angiography-derived index of microcirculatory resistance; DES, drug-eluting stents; DCB, drug-coated balloons; MACE, major adverse cardiovascular events.

The CPFD-IMR measurement was conducted using the software (FlashAngio, Rainmed Ltd., Suzhou, China, Figure 1) as described before (Ai et al., 2020). In brief, a three-dimensional reconstruction of coronary arteries was firstly conducted for the target vessels, followed by the estimation of caIMR by CPFD with a validated method (Li et al., 2020).

After DCB or DES treatment, nitroglycerin was injected. After exposure for 1 s, the contrast agent was injected to IRA at a speed of 4 ml/s. The image recording rate was 15 frames/s, and the contrast agent was stably injected for ≥3 cardiac cycles. Angiographic images of two postures were selected through FlashAngio IMR system (the angle between the included two postures ≥30°), generating a three-dimensional model of the targeted coronary artery. Meanwhile, a 3-dimensional mesh reconstruction of the coronary artery was generated along the vessel path from the inlet to the distal segment of the target vessel. We computed the diastolic flow velocity (Vdiastole) by the TIMI Frame Count Method, i.e., diastolic flow velocity = (contrast passing length)/(diastolic time interval), where contrast passing length was the distance that contrast moves in 3D reconstructed coronary arteries during the period of diastole. The choose of diastolic phase was determined based on the motion of the guide catheter tip.

Using the fully automatic coronary angiography-based FlashAngio IMR system (including FlashAngio IMR console, FlashAngio IMR software, and FlashPressure IMR pressure transducer; Rainmed Ltd., Suzhou, China), a novel physiological parameter, caIMR (unit: mmHg∙s/mm), is calculated as follows:

$$caIMR={\left(P_{a}\right)}_{hyp}\cdot caFFR\cdot L/K\cdot Vdiastole$$ (1)

$$where:caFFR={\left(P_{d}\right)}_{hyp}/{\left(P_{a}\right)}_{hyp}$$ (2)

caFFR is the coronary angiography-derived fractional flow reserve (Ai et al., 2020; Li et al., 2020) that was verified to have a high accuracy compared with wire-based FFR in the previous study, from which we can get:

$${\left(P_{d}\right)}_{hyp}={\left(P_{a}\right)}_{hyp}\cdot caFFR$$ (3)

$$and{\left(P_{d}\right)}_{hyp}={\left(P_{a}\right)}_{hyp}-\Delta P$$ (4)

${\left(P_{a}\right)}_{hyp}$ and ${\left(P_{d}\right)}_{hyp}$ is the mean pressure (unit: mmHg) at the aorta and the distal position at the maximal hyperemia respectively, the subscript “hyp” of ${\left(P_{d}\right)}_{hyp},\;{\left(P_{a}\right)}_{hyp}$ refers to maximal hyperemia state, ΔP is the pressure drop along the coronary artery from the inlet to the most distal location; L is a constant that mimics the length from the inlet to the distal position, labeled with two pressure sensors on a pressure wire ( L=75 mm); ${\text{V}}_{\text{diastole}}$ is the mean flow velocity (unit: mm/s) at diastole, and K, obtained from a previous literature, (Choi et al., 2021) is a constant (K = 1.1) proposed to mimics the flow velocity at the maximal hyperemia:

$$V_{hyp}=K\cdot V_{diastole}$$ (5)

$V_{hyp}$ refers to the mean flow velocity (unit: mm/s) at the distal position at the maximal hyperemia.

According to formulations (Eqs 1, 3, 4), we can deduce:

$$caIMR=\left({\left(P_{a}\right)}_{hyp}-\Delta P\right)*L/K\cdot V_{diastole}$$ (6)

${\left(P_{a}\right)}_{hyp}$ is the maximal hyperemic mean aortic pressure; a pressure sensor was connected to the FlashAngio IMR system to record 3∼8 circles of the pressures wave during the angiography, by averaging the pressure, we can get mean aortic pressure (MAP), based on which ${\left(P_{a}\right)}_{hyp}$ , equals to MAP-MAP*0.2 when MAP ≥95 mmHg and MAP-MAP*0.15 when MAP <95 mmHg (Li et al., 2020).

To compute the pressure drop ΔP , a specially-designed CPFD model was carried out to do the steady-state laminar flow simulation across the stenotic blood vessel, ${\text{V}}_{\text{hyp}}$ calculated from formular (Eq. 5) was used as the inlet boundary condition to solve Navier-Stokes and continuity equations in the FlashAngio IMR system:

$$\nabla \cdot \hat{V}=0$$ (7)

$$\rho \frac{\partial \hat{V}}{\partial t}+\rho \hat{V}\cdot \nabla \hat{V}=-\nabla P+\nabla \cdot \mu \left(\nabla \hat{V}+{\left(\nabla \cdot \hat{V}\right)}^{T}\right)$$ (8)

where $\hat{V}$ , P, ρ, and μ represent the velocity, pressure, blood mass density, and viscosity, respectively. ΔP was obtained by integrating over each grid.

All the CPFD-caIMR metrics were analyzed offline in a core lab (Ai et al., 2020; Li et al., 2020; Choi et al., 2021). Normal reference range for STEMI patients was: CPFD-caIMR ≤ 40U.

### Study Outcomes

Patients were included with a follow-up of at least 12 months. Primary and secondary endpoints were recorded detailly.

The primary endpoint included: 1) difference of CPFD-caIMR between DES and DCB groups and 2) comparing MACEs incidence (cardiovascular or all-cause deaths, non-fatal MI, recurrent unstable angina pectoris (UA), revascularization including target vessel reconstruction, heart failure readmission) between DES and DCB group.

The secondary endpoints included: 1) CPFD-caIMR predicting effects on MACEs and 2) the determination of predictors for MACEs.

### Subgroup Analyses

**FIGURE 2:**
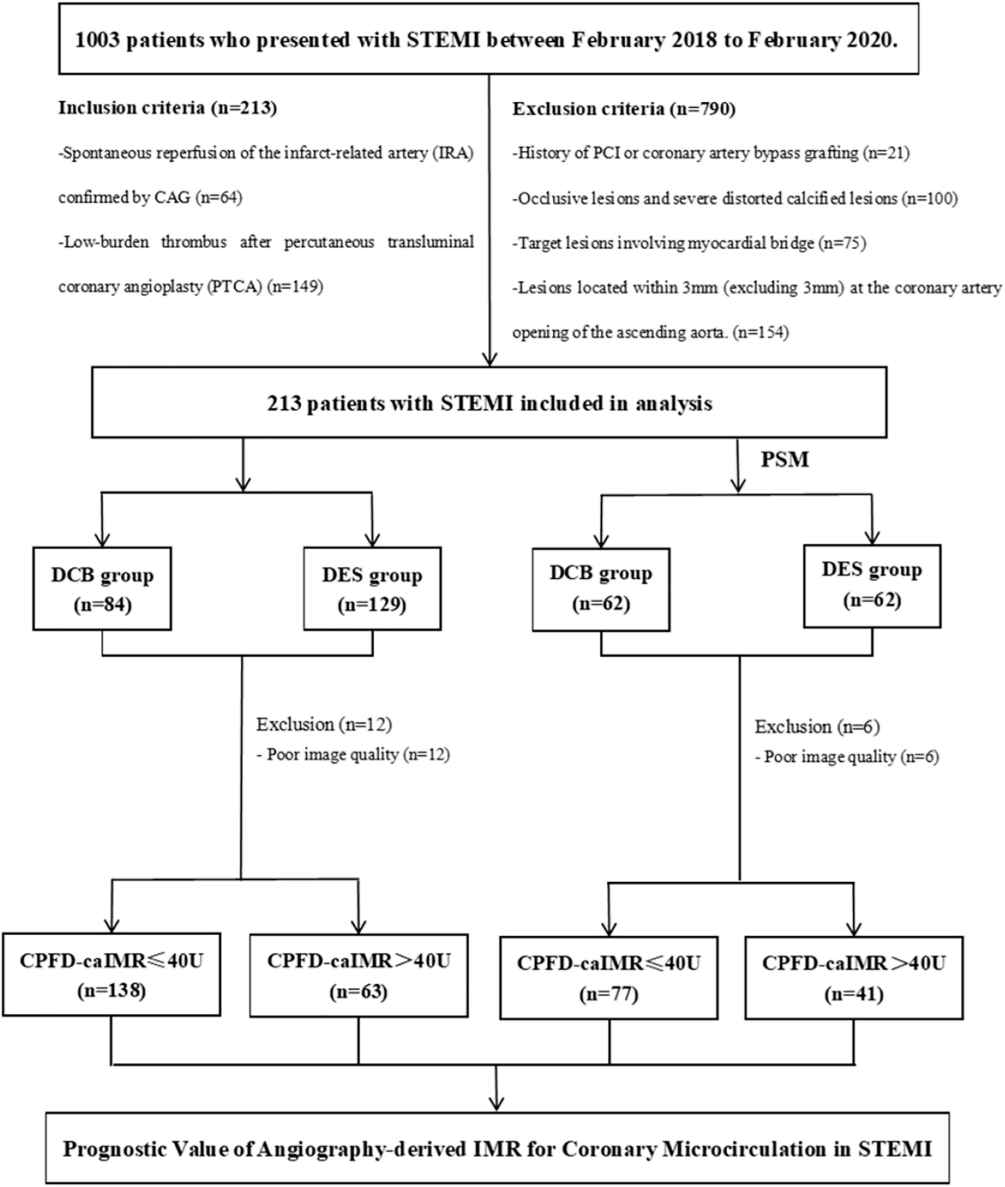
The flow-chart of the study in detail. Abbreviations: STEMI, ST-segment elevation myocardial infarction; PCI, percutaneous coronary intervention; CPFD, computational pressure-fluid dynamics; caIMR, coronary-angiography-derived index of microcirculatory resistance; DES, drug-eluting stents; DCB, drug-coated balloons; MACE, major adverse cardiovascular events.

According to MACEs results, patients were divided into non-MACEs or MACEs groups to find possible predictors including CPFD-caIMR. And according to the cutoff value of CPFD-caIMR of 40U, patients were re-grouped into caIMR ≤ 40U or caIMR >40U groups to determine its predicting value on MACEs. The flow-chart of the study was presented in Figure 2.

### Statistical Analysis

All analyses were conducted after matching baseline characteristics including age, sex, SBP, BMI using PSM. Continuous variables and categorical variables were expressed as mean (standard deviation), median (inter-quartile range) and proportions (%) depending on the circumstance. Student’s or paired t test was used to analyze continuous variables and the chi-square or rank sum was used to analyze categorical variables. Univariate and multivariate logistic regression analysis were used to investigate the predictors of MACEs, reported as odds ratio (OR) and 95% confidence interval (CI). The Receiver Operating characteristic (ROC) curve and Area under curve (AUC) were utilized to evaluate the efficiency of predictors. A two-sided *p* value of <0.05 was considered statistically significant. SPSS (version 23; IBM, Armonk, NY, USA) was used to analyze the relevant data of the study.

## Results

### Study Patients

There were totally 213 patients involved in this retrospectively controlled study, including 84 patients adapting DCB and 129 DES with a follow-up of 1 year. After PSM, DCB and DES groups were comparable regarding characteristics of baseline and PPCI process (Tables 1, 2; Supplementary Tables S1, S2). However, higher baseline left ventricular ejection fraction (LVEF, *p* < 0.01), lower peak high sensitivity troponin T (hsTnT, *p* < 0.01) and less diuretics use (*p* < 0.01) were observed in DCB group than those in DES group. With respect to PPCI process, DES/DCB diameter, pre-dilation released pressure and duration were significantly different in two groups (*p* < 0.01).

**TABLE 1:** Baseline characteristics of the study population.

Variable	Group DES	Group DCB	*p* value	Total
General characteristics	N = 129	N = 84		N = 213
Age, y, Mean (SD)	56.91 (12.27)	60.52 (14.11)	0.05	58.34 (13.11)
Males, n (%)	109 (84.50)	70 (83.33)	0.85	179 (84.04)
Systolic BP, mmHg, Mean (SD)	123.15 (17.93)	131.04 (18.32)	**<0.01**	126.26 (18.45)
Heart rate, /min, Mean (SD)	79.40 (15.92)	75.57 (15.13)	0.08	77.89 (15.69)
Body mass index, kg/m^2^, Mean (SD)	25.94 (4.74)	25.06 (3.22)	0.14	25.59 (4.22)
Hypertension, n (%)	68 (52.71)	37 (44.05)	0.26	105 (49.30)
Diabetes, n (%)	19 (14.73)	19 (22.62)	0.15	38 (17.84)
Smoking, n (%)	68 (52.71)	37 (44.05)	0.26	105 (49.30)
Time from symptom to balloon, hours, Mean (SD)	6.56 (3.44)	7.30 (2.98)	0.11	6.85 (3.28)
Killip level, n (%)				
I	116 (89.92)	78 (92.86)	0.33	194 (91.08)
II	8 (6.20)	4 (4.76)		12 (5.63)
III	2 (1.55)	2 (2.38)		4 (1.89)
IV	3 (2.33)	0		3 (1.41)
Baseline LVEF and biomarkers				
LVEF, %, Mean (SD)	49.13 (9.88)	56.54 (7.56)	**<0.01**	52.05 (9.72)
Peak hsTnT, ng/L, Median (IQR)	3543.00 (4483.00)	1636.00 (4371.75)	**<0.01**	2847.00 (4656.50)
Peak CK-MB, ng/L, Median (IQR)	104.91 (163.25)	71.85 (105.09)	0.11	88.79 (144.67)
CRP, Mean (SD)	12.49 (11.26)	10.70 (9.08)	0.22	11.78 (10.47)
Serum Creatinine, umol/L, Mean (SD)	69.12 (13.36)	66.93 (19.18)	0.33	68.26 (15.91)
LDL-C, mmol/L, Mean (SD)	2.72 (0.93)	2.50 (0.81)	0.08	2.63 (0.89)
Medication, n (%)				
Asprin	124 (100)	89 (100)	NA	213 (100)
P2Y12 inhibitors	124 (100)	89 (100)	NA	213 (100)
Statins	127 (98.45)	81 (96.43)	0.39	208 (97.65)
Beta-blocker	117 (90.70)	67 (79.76)	**0.03**	184 (86.38)
RAASI	97 (75.19)	54 (64.29)	0.09	151 (70.89)
IV diuretics	61 (47.29)	17 (20.24)	**<0.01**	78 (36.62)

DES, drug-eluting stents; DCB, drug-coated balloons; SD, standard deviation; IQR, inter-quartile range; BP, blood pressure; PCI, percutaneous coronary intervention; AMI, acute myocardial infarction; LVEF, left ventricular ejection fraction; hsTnT, high sensitivity troponin T; CK-MB, MB isoenzyme of creatine kinase; CRP, C-reactive protein; LDL-C, low-density lipoprotein cholesterol; RAASI, renin-angiotensin-aldosterone system inhibitor; IV diuretics, Intravenous diuretics; NA, not available.

**TABLE 2:** Characteristics of PPCI process of the study population.

Variable	Group DES	Group DCB	*p* value	Total
	N = 129	N = 84		N = 213
CPFD-caIMR, Mean (SD)^a^	36.49 (21.04)	34.23 (23.91)	0.48	35.58 (22.21)
CPFD-caIMR>40, n (%)^a^	41 (34.17)	22 (27.16)	0.29	63 (31.34)
Time from Door to balloon, minutes, Mean (SD)	70.36 (14.16)	62.18 (16.44)	<0.01	67.13 (15.59)
Criminal vessel, n (%)				
Left anterior descending	59 (45.70)	31 (36.90)	0.08	90 (42.30)
Left circumflex	17 (13.18)	21 (25.00)		38 (17.80)
Right coronary artery	53 (41.10)	32 (38.10)		85 (39.90)
Multi coronary artery lesions, n (%)	81 (62.79)	51 (60.71)	0.77	132 (61.97)
IABP, n (%)	3 (2.33)	0	0.28	3 (1.41)
Pre-dilated balloon diameter, mm, Mean (SD)	2.16 (0.28)	2.64 (0.57)	<0.01	2.35 (0.48)
Pre-dilated balloon pressure, atm, Mean (SD)	9.69 (2.04)	9.69 (2.35)	0.17	9.69 (2.17)
DES/DCB diameter, mm, Mean (SD)	3.09 (0.44)	2.74 (0.55)	0.01	2.95 (0.51)
DES/DCB length, mm, Mean (SD)	27.57 (6.61)	24.63 (5.59)	0.14	26.41 (6.38)
DES/DCB dilation released pressure, atm, Mean (SD)	11.74 (2.35)	9.56 (2.47)	0.76	10.88 (2.62)
DES/DCB dilation duration, second, Mean (SD)	11.01 (8.28)	67.80 (17.82)	<0.01	33.40 (30.65)

a DES group included 121 patients; DCB groups included 80 patients.

PPCI, primary percutaneous coronary intervention; caIMR, coronary-angiography-derived index of microcirculatory resistance; DES, drug-eluting stents; DCB, drug-coated balloons; SD, standard deviation; IABP, intra-aortic balloon pump; NA, not available.

### Primary Endpoints

 After PPCI, CPFD-caIMR was calculated and found no significance between DCB and DES groups both before and after PSM (before PSM: DES 36.49 (21.04) vs. DCB 34.23 (23.91), *p* = 0.48; after PSM: DES 38.68 (22.66) vs. DCB 35.86 (24.89), *p* = 0.52, Table 2; Supplementary Table S2). The microvascular injury evaluation was still similar between two groups when re-grouped by whether CPFD-caIMR > 40U or not (DES vs. DCB: 34.17% vs. 27.16%, *p* = 0.29).

**FIGURE 3:**
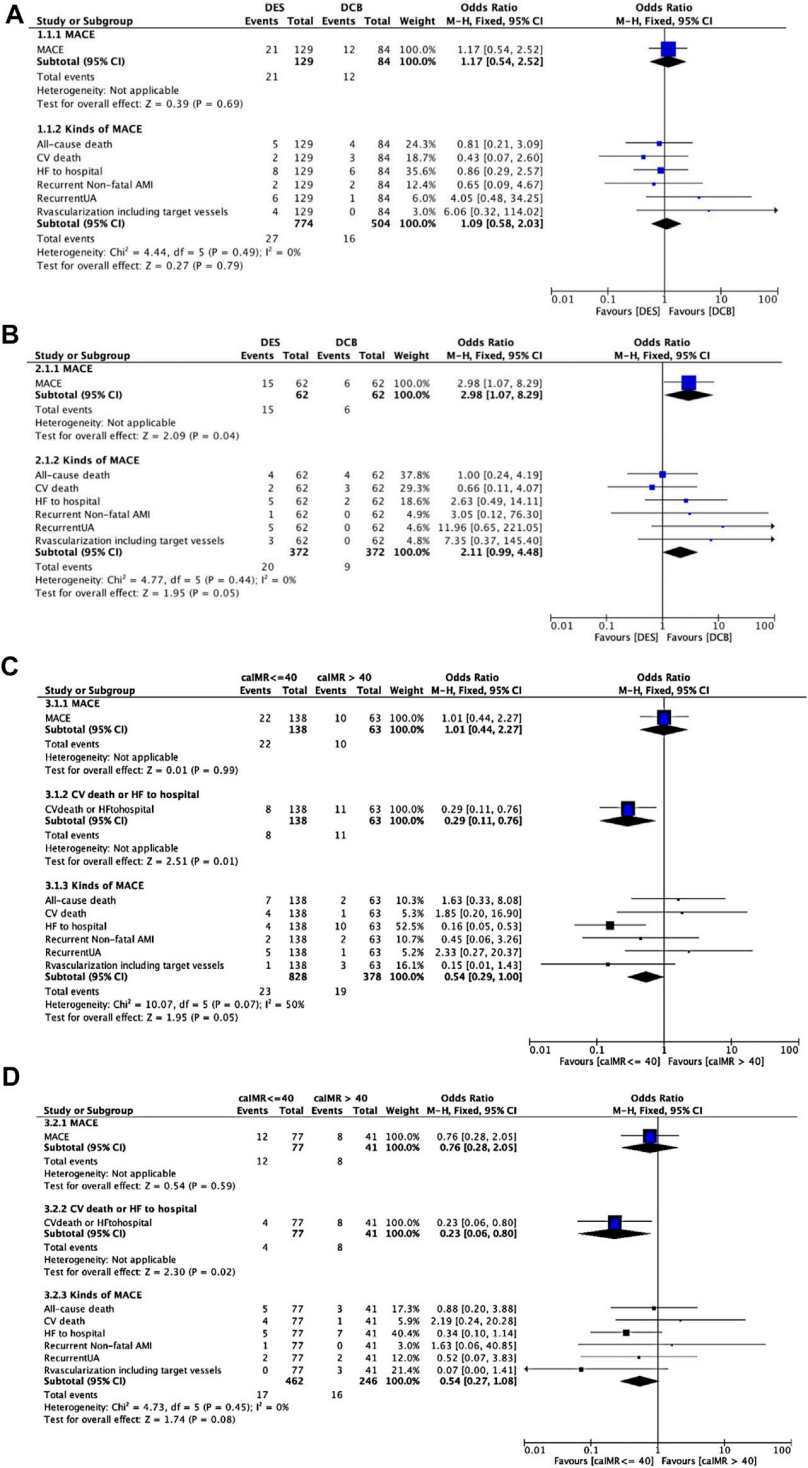
MACEs comparing DES and DCB groups. **(A).** MACEs comparing DES and DCB groups before propensity score matching. **(B)**. MACEs comparing DES and DCB groups after propensity score matching. **(C)**. MACEs comparing caIMR ≤ 40U and caIMR >40U in group DES. **(D)**. MACEs comparing caIMR ≤ 40U and caIMR >40U in group DCB. Abbreviations: DES, drug-eluting stents; DCB, drug-coated balloons; caIMR, coronary-angiography-derived index of microcirculatory resistance; MACE, major adverse cardiovascular events; CV, cardiovascular; HF, heart failure; AMI, acute myocardial infarction; UA, unstable angina; CI, confidence interval.

The statistics for MACEs were presented in Figure 3. MACEs occurred in 21 patients (16.28%) in the DES group and 12 patients (14.29%) in the DCB group (OR 1.17, 95% CI: 0.54 to 2.52, *p* = 0.69, Figure 3A). DCB was similar to DES as regards cardiovascular (CV) deaths (*p* = 0.35), non-CV deaths (*p* = 0.56), non-fatal MI (*p* = 0.66), revascularization (*p* = 0.23), heart failure readmission (*p* = 0.79) and recurrent UA (*p* = 0.20). After PSM (Figure 3B), MACEs were less in DCB than in DES group (DES vs. DCB: OR: 2.98, 95% CI: 1.07 to 8.29, *p* = 0.04) but the incidence of CV deaths (DES vs. DCB: OR: 0.66, 95% CI: 0.11 to 4.07, *p* = 0.65) were the same between the two groups.

### Secondary Endpoints and Subgroup Analysis

Patients were assigned into the MACEs (33 cases) and non-MACEs (180 cases) groups for logistic regression analysis. From ultivariate analysis (Tables 3, 4; Supplementary Tables S3, S4), MACEs group consisted of patients with older age (62.76 (13.50) vs. 57.53 (12.92) years, *p* = 0.04), longer time from symptom to balloon (STOB) (9.47 (2.48) vs. 6.37 (3.18) hours, *p* < 0.01), longer time from door to balloon (DTOB) (87.36 (6.42) vs. 63.42 (13.82) minutes, *p* < 0.01), decreasing LVEF (46.52 (9.48) vs. 53.07 (9.44) %, *p* < 0.01) and higher peak MB isoenzyme of creatine kinase (CK-MB, 220.00 (153.85) vs. 67.96 (100.12) 218.62 ng/L, *p* < 0.01). After PSM, male (*p* < 0.01), LVEF (*p* = 0.01), peak CK-MB (*p* < 0.01), time from STOB (*p* < 0.01), time from DTOB (*p* < 0.01), DCB intervention (*p* = 0.03), pre-dilated balloon diameter (*p* = 0.03), duration of DCB/DES dilation (*p* = 0.01) and lengths of DCB/DES (*p* = 0.01) were associated with MACEs.

**TABLE 3:** Baseline characteristics of the study population grouped by MACEs.

Variable	N-MACE	MACE	*p* value
General characteristics	N = 180	N = 33	
Age, y, Mean (SD)	57.53 (12.92)	62.76 (13.50)	**0.04**
Males, n (%)	155 (86.11)	24 (72.73)	0.07
Systolic BP, mmHg, Mean (SD)	125.26 (17.71)	131.73 (21.55)	0.06
Heart rate, /min, Mean (SD)	78.47 (15.98)	74.73 (13.78)	0.21
Body mass index, kg/m^2^, Mean (SD)	25.74 (4.33)	24.80 (3.51)	0.24
Hypertension, n (%)	85 (47.22)	20 (60.61)	0.19
Diabetes, n (%)	33 (18.33)	5 (15.15)	0.81
Smoking, n (%)	88 (48.89)	17 (51.52)	0.85
Time from symptom to balloon, hours, Mean (SD)	6.37 (3.18)	9.47 (2.48)	**<0.01**
Killip level, n (%)			
I	164 (91.11)	30 (90.91)	0.84
II	10 (5.56)	2 (6.06)	
III	3 (1.67)	1 (3.03)	
IV	3 (1.67)	0	
Baseline LVEF and biomarkers			
LVEF, %, Mean (SD)	53.07 (9.44)	46.52 (9.48)	**<0.01**
Peak hsTnT, ng/L, Median (IQR)	2934.50 (4872.75)	3011.44 (4493.50)	0.32
Peak CK-MB, ng/L, Median (IQR)	67.96 (100.12)	220.00 (153.85)	**<0.01**
CRP, Mean (SD)	11.90 (10.67)	11.15 (9.39)	0.71
Serum Creatinine, umol/L, Mean (SD)	68.79 (15.90)	65.33 (15.844)	0.25
LDL-C, mmol/L, Mean (SD)	2.61 (0.90)	2.74 (0.86)	0.45
Medication, n (%)			
Asprin	180 (100)	33 (100)	NA
P2Y12 inhibitors	180 (100)	33 (100)	NA
Statins	175 (97.22)	33 (100)	0.99
Beta-blocker	154 (85.56)	30 (90.91)	0.58
RAASI	97 (53.88)	54 (64.29)	0.09
IV diuretics	62 (34.44)	16 (48.48)	0.17

DES, drug-eluting stents; DCB, drug-coated balloons; SD, standard deviation; IQR, inter-quartile range; BP, blood pressure; PCI, percutaneous coronary intervention; AMI, acute myocardial infarction; LVEF, left ventricular ejection fraction; hsTnT, high sensitivity troponin T; CK-MB, MB isoenzyme of creatine kinase; CRP, C-reactive protein; LDL-C, low-density lipoprotein cholesterol; RAASI, renin-angiotensin-aldosterone system inhibitor; IV diuretics, Intravenous diuretics; NA, not available.

**TABLE 4:** Characteristics of PPCI process of the study population grouped by MACEs.

Variable	N-MACE	MACE	*p* value
	N = 180	N = 33	
DCB Intervention, n (%)	72 (40.00)	12 (36.36)	0.85
caIMR, Mean (SD)^a^	35.70 (22.39)	34.92 (21.55)	0.85
caIMR>40, n (%)^a^	53 (31.36)	10 (31.25)	0.99
Door to balloon, minutes, Mean (SD)	63.42 (13.82)	87.36 (6.42)	**<0.01**
Criminal vessel, n (%)			
Left anterior descending	72 (40.00)	18 (54.50)	0.28
Left circumflex	34 (18.90)	4 (12.12)	
Right coronary artery	74 (41.10)	11 (33.33)	
Multi coronary artery lesions, n (%)	115 (63.89)	17 (51.52)	0.18
IABP, n (%)	2 (1.11)	1 (3.03)	0.39
Pre-dilated balloon diameter, mm, Mean (SD)	2.36 (0.49)	2.28 (0.41)	0.38
Pre-dilated balloon pressure, atm, Mean (SD)	9.76 (2.25)	9.33 (1.63)	0.21
DES/DCB diameter, mm, Mean (SD)	2.96 (0.51)	2.88 (0.50)	0.39
DES/DCB length, mm, Mean (SD)	26.15 (6.52)	27.85 (5.38)	0.16
DES/DCB dilation released pressure, atm, Mean (SD)	10.83 (2.65)	11.12 (2.48)	0.56
DES/DCB dilation duration, second, Mean (SD)	34.11 (31.28)	29.55 (27.11)	0.43

a DES group included 121 patients; DCB groups included 80 patients.

PPCI, primary percutaneous coronary intervention; caIMR, coronary-angiography-derived index of microcirculatory resistance; DES, drug-eluting stents; DCB, drug-coated balloons; SD, standard deviation; IABP, intra-aortic balloon pump; NA, not available.

**FIGURE 4:**
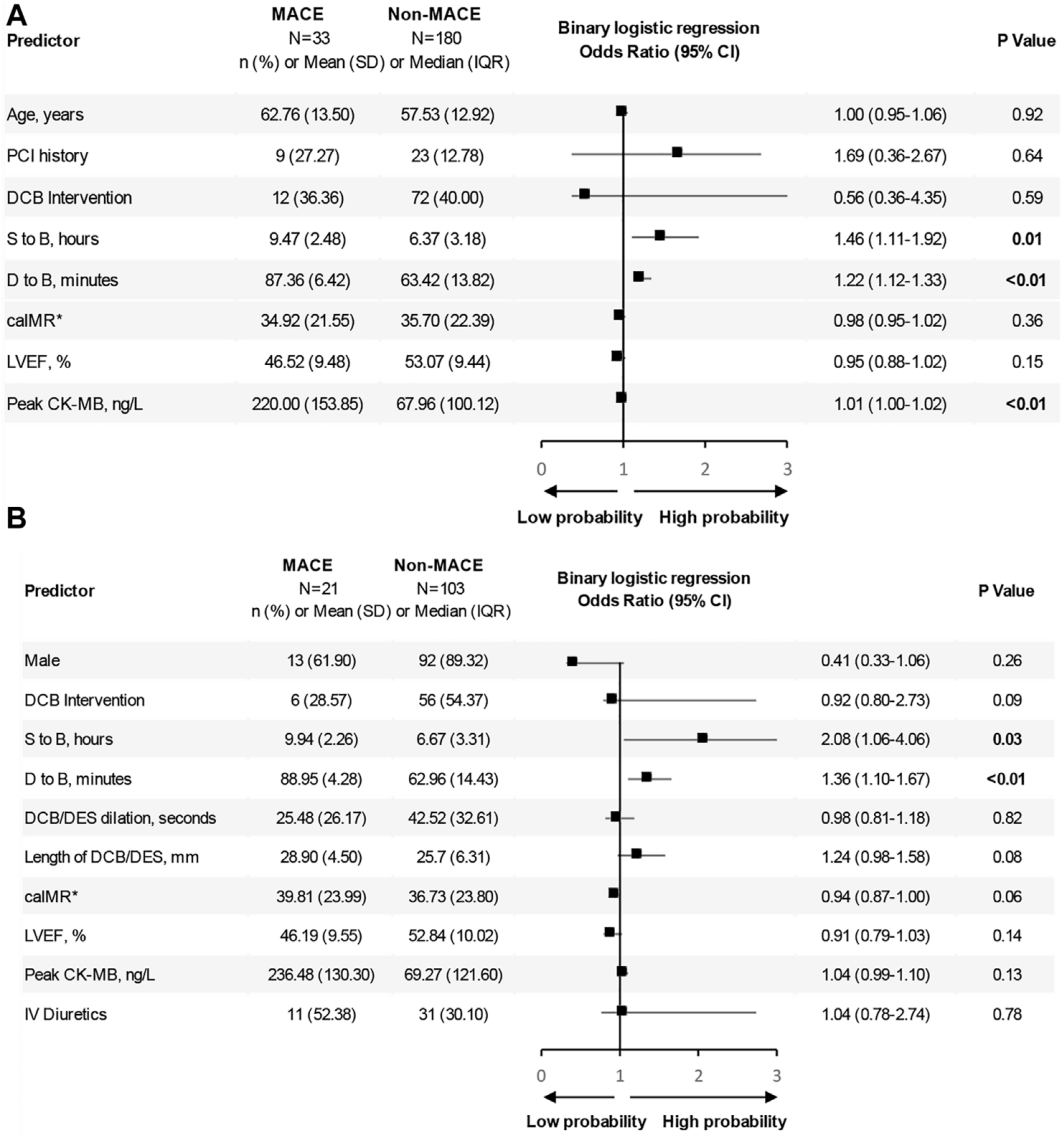
Binary logistic regression analysis. Shown are odds ratios for MACEs among patients. **(A)** Binary logistic regression analysis before propensity score matching. Shown are odds ratios for MACEs among patients before propensity score matching. The size of the square corresponds to the number of patients in two groups. **(B)** Binary logistic regression analysis after propensity score matching. Shown are odds ratios for MACEs among patients after propensity score matching. The size of the square corresponds to the number of patients in two groups. Abbreviations: MACE, major adverse cardiovascular events; PCI, percutaneous coronary intervention; DCB, drug-coated balloons; DES, drug-eluting stents; S to B, time from symptom to balloon; D to B, time from door to balloon; caIMR, coronary-angiography-derived index of microcirculatory resistance; LVEF, Left ventricular ejection fraction; CK-MB, MB isoenzyme of creatine kinase; IV diuretics, Intravenous diuretics; SD, standard deviation; IQR, inter-quartile range; CI, confidence interval.

**FIGURE 5:**
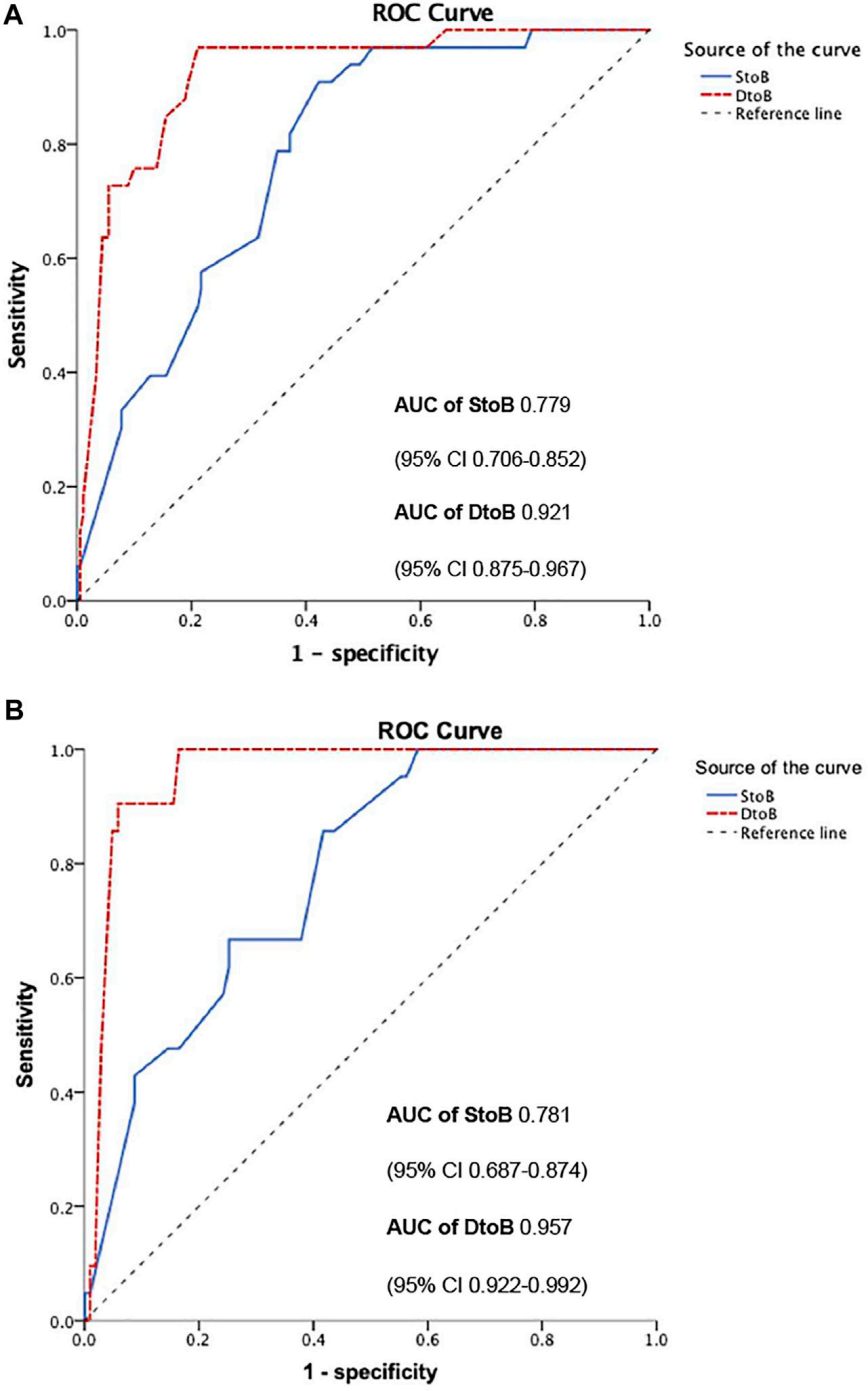
ROC curve of STOB and DTOB predicting MACEs before **(A)** and after **(B)** propensity score matching. Abbreviations: S to B, time from symptom to balloon; D to B, time from door to balloon; ROC, Receiver Operating characteristic; AUC, Area under curve.

In multi-variate analysis (Figure 4), only time from STOB (after PSM: OR: 2.08, 95% CI: 1.06 to 4.06, *p* = 0.03) and time from DTOB (after PSM: OR: 1.36, 95% CI: 1.10 to 1.67, *p* < 0.01) were highly related to MACEs both before and after PSM. After PSM, the logistic model was statistically significant (χ2 = 87.91, *p* = 0.000) and fitted well. Among 10 independent variables involved in the model, time from STOB and time from DTOB were statistically significant. The probability of MACEs of patients with a long time from STOB and DTOB was 2.08 times and 1.36 times that of patients with a short time from STOB and DTOB (Figure 4), respectively. The ROC curve of STOB and DTOB was presented in Figure 5. The sensitivity and specificity for predicting MACEs were 85.0% and 99.0%, respectively.

### The Relationship of CPFD-caIMR and Prognosis

When we assigned patients into CPFD-caIMR ≤ 40U and CPFD-caIMR > 40U groups (Choi et al., 2021), characteristics were not significantly different between two groups, except the criminal vessels (*p* < 0.05, Supplementary Tables S5, S6). Although the incidence of revascularization including target vessels was related to high CPFD-caIMR (r = 0.22, *p* = 0.02) when re-grouped by the cut-off value of CPFD-caIMR of 40 after PSM, this effect was no longer significant from multivariate analysis.

**FIGURE 6:**
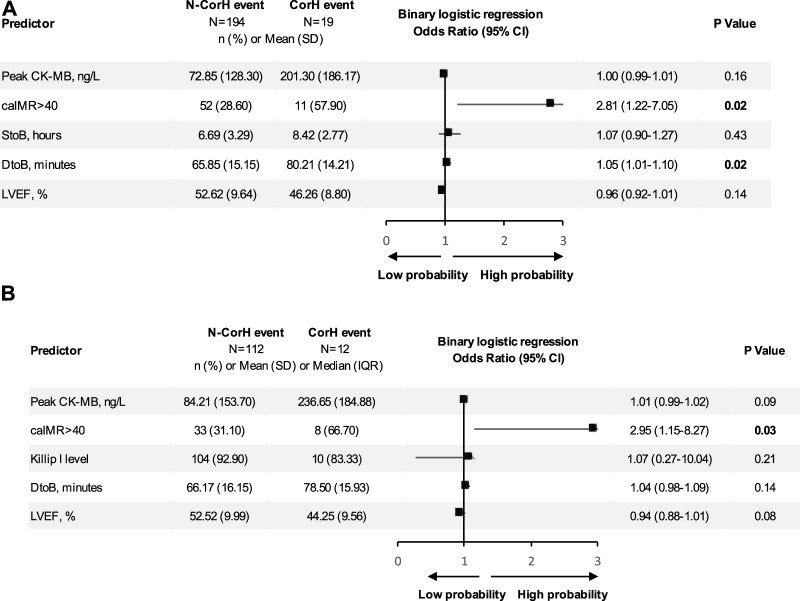
Binary logistic regression analysis. Shown are odds ratios for CorH event among patients. **(A)** Binary logistic regression analysis of predicting the event group including CV deaths or heart failure readmission (CorH) before propensity score matching. Shown are odds ratios for CorH event among patients before propensity score matching. The size of the square corresponds to the number of patients in two groups. **(B)** Binary logistic regression analysis of predicting the event group including CV deaths or heart failure readmission (CorH) after propensity score matching. Shown are odds ratios for CorH event among patients after propensity score matching. The size of the square corresponds to the number of patients in two groups. Abbreviations: S to B, time from symptom to balloon; D to B, time from door to balloon; caIMR, coronary-angiography-derived index of microcirculatory resistance; LVEF, Left ventricular ejection fraction; CK-MB, MB isoenzyme of creatine kinase; SD, standard deviation; IQR, inter-quartile range; CI, confidence interval.

**FIGURE 7:**
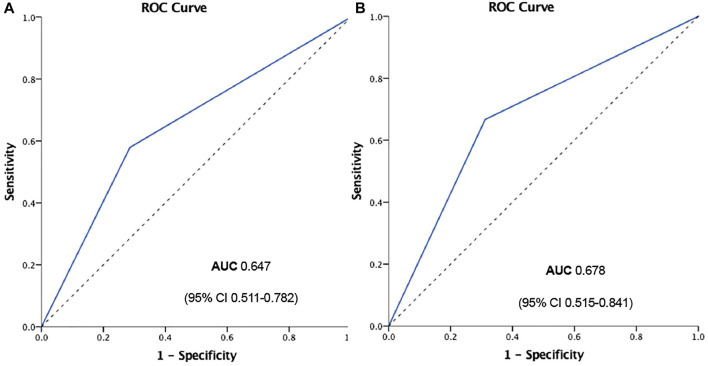
ROC curve of CPFD-caIMR predicting the event group including CV deaths or heart failure readmission (CorH) before **(A)** and after **(B)** propensity score matching. Abbreviations: CPFD, computational pressure-fluid dynamics; caIMR, coronary-angiography-derived index of microcirculatory resistance; CV, cardiovascular; ROC, Receiver Operating characteristic; AUC, Area under curve.

However, when we separately analyzed every kind of event (Figures 3C,D), CPFD-caIMR showed a significant relation with the event group including CV deaths or heart failure readmission (C or H, OR 2.81, 95% CI: 1.22 to 7.05, *p* = 0.02). After PSM, the effects of CPFD-caIMR > 40U predicting incidence of events of C or H remained significant (OR 2.95, 95% CI: 1.15 to 8.27, *p* = 0.03, Figures 6, 7).

## Discussion

Restoring myocardial blood flow of IRA in STEMI patients is of importance in decreasing mortality, but it has been reported that recurrent angina after PCI happened to 20%–60% of patients (Alexander et al., 2016). Coronary microvascular injury as one of the main reasons may be a new therapeutic target (De Waha et al., 2017; Bil et al., 2018). Non-invasive imaging modalities such as cardiac magnetic resonance (MRI) was more recognized for microvascular injury evaluation, but not available at the cardiac catheterization laboratory during PPCI.

IMR has been widely studied as an invasive physiological index of microvascular injury after PPCI. Some studies have found that IMR is significantly correlated with prognosis (Cuculi et al., 2014; Fahrni et al., 2017; De Maria et al., 2019). The traditional IMR measurement is based on thermodilution-pressure wire. The risk for manipulation of guiding wire and hyperemic agent has limited its application in the clinical uses, added with the operation time over 40 min, it results in a research tool in the laboratory (Choi et al., 2021). Except for MRI and positron emission tomography, more noninvasive assessments for microvascular dysfunction have been brought to the public. A novel index of microcirculatory resistance that based on Digital Imaging and Communications in Medicine (DICOM) angiography images, computational fluid dynamics (CPFD) model and aortic pressure waves, caIMR has been proved to have a high accuracy compared with wire based IMR. By CPFD method, caIMR was calculated within 1min and the whole measuring process needs less than 5 min, which enables the diagnosis of microcirculatory dysfunction made synchronously with angiographic surgery (Collet et al., 2018). Noninvasive measurement has attracted more and more attention (Ai et al., 2020). Recent researches have confirmed that CPFD-caIMR is a promising alternative method of IMR to evaluate the prognosis of STEMI patients, since wired and hyperemic agent based IMR is not appropriate in the perioperative period for revascularization after STEMI (Abuouf et al., 2021). Our study took advantage of CPFD-caIMR as post-operative index and no significant difference of effects on microvascular injury comparing DCB with DES. Similarly, the cardiovascular outcomes of DCB group were also comparable to those of DES group no matter before or after PSM, suggesting possibly similar effects of DCB and DES.

PPCI remains the main means for rapid recovery of coronary blood flow, but its side-effect of bringing thrombosis is still worrying (Her et al., 2021). The advantage of DCB is to avoid the implantation of metal stents and minimize potential long-term safety problems. This single center, retrospectively controlled trial of DCB and DES in STEMI patients undergoing PPCI, whose lesions were spontaneous reperfusion of the IRA confirmed by CAG or low-burden thrombus after PTCA, showed that there was no significant difference of general characteristics between DCB and DES after PSM. And we found similar CPFD-caIMR between DCB and DES treatment but the incidence of MACEs was less in DCB group than that in DES group.

The using of DCB has been proved to be effective for ISR, and thus recommended by the European, German, and Asia Pacific consensus group (Aboyans et al., 2018; Her et al., 2021). In recent years, with the concept of “intervention without implantation” increasingly rooting and spreading, the application of DCB has gradually expanded to *de novo* lesions in RCTs even clinical practice (Steigen et al., 2006; Wöhrle and Werner, 2013; Her et al., 2016; Shin et al., 2016; Jeger et al., 2018). Hitherto, DCB alone has been proved similar effects with DES implantation in STEMI patients in terms of proximal and middle lesions in PAPPA research, with 5% occurrence of MACEs within 1 year. (Vos et al., 2014). The similar results manifested in Gobić et al. (2017) study and the late lumen loss of DCB at 6 months was better than that of DES . However, there was a positive result supporting DCB for reducing MACEs from our study after PSM eliminating the effects of confounders. There is no doubt that this positive finding benefits the application of DCB in clinical practice and provide some evidence of DCB use in PPCI of STEMI patients. Part of the reasons, there was a much higher drug concentration in the vascular wall after DCB use than after DES implantation (Vogt et al., 2004; Speck et al., 2012) resulting in cytostasis as well as mitotic and post-mitotic arrest (Axel et al., 1997). Kleber et al. (2015) showed that DCB had a positive remodeling effect, and more lumen was obtained in the late stage. This made up for the disadvantage of postoperative residual stenosis in DCB group, and explained another possible reason why prognosis in DCB group was better than DES group.

We also tried to determine predictors of MACEs including DCB intervention and CPFD-caIMR. In fact, high burden of thrombus and micro embolism were one of the main reasons affecting microvascular injury and CPFD-caIMR value (Gupta and Gupta, 2016), but patients in our study scarcely had the situations above mentioned. Lesions of patients all included spontaneous reperfusion of the IRA confirmed by CAG or low-burden thrombus after PTCA. The PPCI performance was well-prepared and the blood flow of criminal vessels resumed TIMI Level 3, so CPFD-caIMR value was not to such an extent as to be significantly different. However, CPFD-caIMR > 40U well predicted the combined events group including CV deaths or heart failure readmission. This point also corresponded to previous findings (Choi et al., 2021). Clinically, we need to pay more attention to CPFD-caIMR guided-treatment strategy and prognosis management to improve the quality of life of patients.

There are some limitations in this study. Firstly, this study was a retrospective controlled trial but not RCT with limited sample size. These contributed to differences of baseline characteristics, but PSM was conducted to deal with the study design of the observational study and to make the results more credible. Secondly, though we found DCB was not inferior to DES in MACEs in STEMI patients with PPCI, further large prospective RCTs were necessary to confirm this conclusion due to the unchangeable limitations of small sample size and short follow-up time. Our study data just provided some evidence about DCB clinical application.

## Conclusion

Microvascular injury evaluation based on CPFD-caIMR was similar between DES and DCB treatments. The DCB strategy during PPCI in STEMI patients may be a safe and feasible alternative strategy for DES treatment for less MACEs, in patients with spontaneous reperfusion of the IRA confirmed by CAG or low-burden thrombus after PTCA. CPFD-CaIMR is a promising alternative method of IMR, which can be used to evaluate the prognosis of STEMI patients with PPCI who will possibly experience CV deaths or heart failure readmission in future.

## Data Availability

The raw data supporting the conclusion of this article will be made available by the authors, without undue reservation.

## Supplementary Material

Supplementary Table S1 Baseline characteristics of the study population after propensity score matching.

Supplementary Table S2 Characteristics of PPCI process after propensity score matching.

Supplementary Table S3 Baseline characteristics of the study population re-grouped by MACEs after propensity score matching.

Supplementary Table S4 Characteristics of PPCI process re-grouped by MACEs after propensity score matching.

Supplementary Table S5 Baseline characteristics of the study population grouped by caIMR.

Supplementary Table S6 Characteristics of PPCI process of the study population grouped by caIMR.

## Glossary

AUC: Area under curve
caFFR: coronary angiography-derived fractional flow reserve
CAG: coronary angiography
CI: confidence interval
CK-MB: MB isoenzyme of creatine kinase
CCU: coronary care unit
CPFD: computational pressure-fluid dynamics
CPFD-caIMR: computational pressure-fluid dynamics derived index of microcirculatory resistance, applied to coronary angiography
CV: cardiovascular
DAPT: dual antiplatelet therapy
DCB: Drug-coated balloon
DES: Drug-eluting stent
DTOB: door to balloon
hsTnT: high sensitivity troponin T
hyp: hyperemia flow velocity
IMR: Index of microcirculatory resistance
IRA: infarct-related artery
ISR: in-stent restenosis
LVEF: left ventricular ejection fraction
MACEs: major adverse cardiovascular events
MAP: Mean arterial pressure
OR: odds ratio
PPCI: primary percutaneous coronary intervention
PSM: propensity score matching
RCTs: randomized controlled trials
ROC: Receiver Operating characteristic
STEMI: ST-segment elevation myocardial infarction
STOB: symptom to balloon
TIMI: Thrombolysis in myocardial infarction
UA: unstable angina pectoris

## References

[B1] Aboyans V. Ricco J. B. Bartelink M. E. L. Björck M. Brodmann M. Cohnert T. (2018). 2017 ESC Guidelines on the Diagnosis and Treatment of Peripheral Arterial Diseases, in Collaboration with the European Society for Vascular Surgery (ESVS): Document Covering Atherosclerotic Disease of Extracranial Carotid and Vertebral, Mesenteric, Renal, Upper and Lower Extremity arteriesEndorsed by: the European Stroke Organization (ESO)The Task Force for the Diagnosis and Treatment of Peripheral Arterial Diseases of the European Society of Cardiology (ESC) and of the European Society for Vascular Surgery (ESVS). Eur. Heart J. 39, 763–816. 10.1093/eurheartj/ehx095 28886620

[B2] Abuouf Y. AlBadawi M. Ookawara S. Ahmed M. (2021). Effect of Guidewire Insertion in Fractional Flow Reserve Procedure for Real Geometry Using Computational Fluid Dynamics. Biomed. Eng. Online 20, 95. 10.1186/s12938-021-00935-y 34583689 PMC8479905

[B3] Ai H. Feng Y. Gong Y. Zheng B. Jin Q. Zhang H.-P. (2020). Coronary Angiography-Derived Index of Microvascular Resistance. Front. Physiol. 11, 605356. 10.3389/fphys.2020.605356 33391020 PMC7772433

[B4] Alekseeva Y. V. Vyshlov E. V. Pavlyukova E. N. Ussov V. Y. Markov V. A. Ryabov V. V. (2021). Impact of Microvascular Injury Various Types on Function of Left Ventricular in Patients with Primary Myocardial Infarction with ST Segment Elevation. Kardiologiia 61, 23–31. 10.18087/cardio.2021.5.n1500 34112072

[B5] Alexander K. P. Weisz G. Prather K. James S. Mark D. B. Anstrom K. J. (2016). Effects of Ranolazine on Angina and Quality of Life after Percutaneous Coronary Intervention with Incomplete Revascularization. Circulation 133, 39–47. 10.1161/CIRCULATIONAHA.115.019768 26555329

[B6] Axel D. I. Kunert W. Göggelmann C. Oberhoff M. Herdeg C. Küttner A. (1997). Paclitaxel Inhibits Arterial Smooth Muscle Cell Proliferation and Migration *In Vitro* and *In Vivo* Using Local Drug Delivery. Circulation 96, 636–645. 10.1161/01.cir.96.2.636 9244237

[B7] Bil J. Pietraszek N. Pawlowski T. Gil R. J. (2018). Advances in Mechanisms and Treatment Options of MINOCA Caused by Vasospasm or Microcirculation Dysfunction. Cpd 24, 517–531. 10.2174/1381612824666180108121253 29308736

[B8] Carrick D. Haig C. Ahmed N. Carberry J. Yue May V. T. McEntegart M. (2016). Comparative Prognostic Utility of Indexes of Microvascular Function Alone or in Combination in Patients with an Acute ST-Segment-Elevation Myocardial Infarction. Circulation 134, 1833–1847. 10.1161/circulationaha.116.022603 27803036 PMC5131697

[B9] Choi K. H. Dai N. Li Y. Kim J. Shin D. Lee S. H. (2021). Functional Coronary Angiography-Derived Index of Microcirculatory Resistance in Patients with ST-Segment Elevation Myocardial Infarction. JACC Cardiovasc. Interv. 14, 1670–1684. 10.1016/j.jcin.2021.05.027 34353599

[B10] Collet C. Onuma Y. Sonck J. Asano T. Vandeloo B. Kornowski R. (2018). Diagnostic Performance of Angiography-Derived Fractional Flow Reserve: a Systematic Review and Bayesian Meta-Analysis. Eur. Heart J. 39, 3314–3321. 10.1093/eurheartj/ehy445 30137305

[B11] Cuculi F. De Maria G. L. Meier P. Dall'Armellina E. de Caterina A. R. Channon K. M. (2014). Impact of Microvascular Obstruction on the Assessment of Coronary Flow Reserve, Index of Microcirculatory Resistance, and Fractional Flow Reserve after ST-Segment Elevation Myocardial Infarction. J. Am. Coll. Cardiol. 64, 1894–1904. 10.1016/j.jacc.2014.07.987 25444143

[B12] De Luca G. Suryapranata H. Stone G. W. Antoniucci D. Biondi-Zoccai G. Kastrati A. (2008). Coronary Stenting versus Balloon Angioplasty for Acute Myocardial Infarction: a Meta-Regression Analysis of Randomized Trials. Int. J. Cardiol. 126, 37–44. 10.1016/j.ijcard.2007.03.112 17544528

[B13] De Maria G. L. Alkhalil M. Wolfrum M. Fahrni G. Borlotti A. Gaughran L. (2019). Index of Microcirculatory Resistance as a Tool to Characterize Microvascular Obstruction and to Predict Infarct Size Regression in Patients with STEMI Undergoing Primary PCI. JACC Cardiovasc. Imaging 12, 837–848. 10.1016/j.jcmg.2018.02.018 29680355

[B14] De Waha S. Patel M. R. Granger C. B. Ohman E. M. Maehara A. Eitel I. (2017). Relationship between Microvascular Obstruction and Adverse Events Following Primary Percutaneous Coronary Intervention for ST-Segment Elevation Myocardial Infarction: an Individual Patient Data Pooled Analysis from Seven Randomized Trials. Eur. Heart J. 38, 3502–3510. 10.1093/eurheartj/ehx414 29020248

[B15] Fahrni G. Wolfrum M. De Maria G. L. Cuculi F. Dawkins S. Alkhalil M. (2017). Index of Microcirculatory Resistance at the Time of Primary Percutaneous Coronary Intervention Predicts Early Cardiac Complications: Insights from the OxAMI (Oxford Study in Acute Myocardial Infarction) Cohort. Jaha 6, e005409. 10.1161/JAHA.116.005409 29113999 PMC5721736

[B16] Geng Y. Wu X. Liu H. Zheng D. Xia L. (2022). Index of Microcirculatory Resistance: State-Of-The-Art and Potential Applications in Computational Simulation of Coronary Artery Disease. J. Zhejiang Univ. Sci. B 23, 123–140. 10.1631/jzus.B2100425 35187886 PMC8861561

[B17] Gobić D. Tomulić V. Lulić D. Židan D. Brusich S. Jakljević T. (2017). Drug-Coated Balloon versus Drug-Eluting Stent in Primary Percutaneous Coronary Intervention: A Feasibility Study. Am. J. Med. Sci. 354, 553–560. 10.1016/j.amjms.2017.07.005 29208251

[B18] Gupta S. Gupta M. M. (2016). No Reflow Phenomenon in Percutaneous Coronary Interventions in ST-Segment Elevation Myocardial Infarction. Indian Heart J. 68, 539–551. 10.1016/j.ihj.2016.04.006 27543480 PMC4990737

[B19] Her A.-Y. Ann S. H. Singh G. B. Kim Y. H. Okamura T. Garg S. (2016). Serial Morphological Changes of Side-Branch Ostium after Paclitaxel-Coated Balloon Treatment of De Novo Coronary Lesions of Main Vessels. Yonsei Med. J. 57, 606–613. 10.3349/ymj.2016.57.3.606 26996558 PMC4800348

[B20] Her A.-Y. Shin E.-S. Bang L. H. Nuruddin A. A. Tang Q. Hsieh I.-C. (2021). Drug-coated Balloon Treatment in Coronary Artery Disease: Recommendations from an Asia-Pacific Consensus Group. Cardiol. J. 28, 136–149. 10.5603/CJ.a2019.0093 31565793 PMC8105061

[B21] Ho H. H. Tan J. Ooi Y. W. Loh K. K. Aung T. H. Yin N. T. (2015). Preliminary Experience with Drug-Coated Balloon Angioplasty in Primary Percutaneous Coronary Intervention. Wjc 7, 311–314. 10.4330/wjc.v7.i6.311 26131335 PMC4478565

[B22] Ibanez B. James S. Agewall S. Antunes M. J. Bucciarelli-Ducci C. Bueno H. (2018). ESC Guidelines for the Management of Acute Myocardial Infarction in Patients Presenting with ST-Segment Elevation: The Task Force for the Management of Acute Myocardial Infarction in Patients Presenting with ST-Segment Elevation of the European Society of Cardiology (ESC). Eur. Heart J. 39, 119–177. 10.1093/eurheartj/ehx393 28886621

[B23] Jaski B. E. Grigoriadis C. E. Dai X. Meredith R. D. Ortiz B. C. Stouffer G. A. (2016). Factors Associated with Ineligibility for PCI Differ between Inpatient and Outpatient ST-Elevation Myocardial Infarction. J. Interv. Cardiol. 29, 363–369. 10.1111/joic.12306 27364755

[B24] Jeger R. V. Farah A. Ohlow M. A. Mangner N. Möbius-Winkler S. Leibundgut G. (2018). Drug-coated Balloons for Small Coronary Artery Disease (BASKET-SMALL 2): an Open-Label Randomised Non-inferiority Trial. Lancet 392, 849–856. 10.1016/S0140-6736(18)31719-7 30170854

[B25] Kleber F. X. Schulz A. Waliszewski M. Hauschild T. Böhm M. Dietz U. (2015). Local Paclitaxel Induces Late Lumen Enlargement in Coronary Arteries after Balloon Angioplasty. Clin. Res. Cardiol. 104, 217–225. 10.1007/s00392-014-0775-2 25349065

[B26] Li J. Gong Y. Wang W. Yang Q. Liu B. Lu Y. (2020). Accuracy of Computational Pressure-Fluid Dynamics Applied to Coronary Angiography to Derive Fractional Flow Reserve: FLASH FFR. Cardiovasc Res. 116, 1349–1356. 10.1093/cvr/cvz289 31693092

[B27] Neumann F. J. Sousa-Uva M. Ahlsson A. Alfonso F. Banning A. P. Benedetto U. (2019). 2018 ESC/EACTS Guidelines on Myocardial Revascularization. Eur. Heart J. 40, 87–165. 10.1093/eurheartj/ehy394 30165437

[B28] Niehe S. R. Vos N. S. Van Der Schaaf R. J. Amoroso G. Herrman J. R. Patterson M. S. (2022). Two-Year Clinical Outcomes of the REVELATION Study: Sustained Safety and Feasibility of Paclitaxel-Coated Balloon Angioplasty versus Drug-Eluting Stent in Acute Myocardial Infarction. J. Invasive Cardiol. 34, E39–E42. 34792482 10.25270/jic/20.00741

[B29] Nijhoff F. Agostoni P. Belkacemi A. Nathoe H. M. Voskuil M. Samim M. (2015). Primary Percutaneous Coronary Intervention by Drug-Eluting Balloon Angioplasty: the Nonrandomized Fourth Arm of the DEB-AMI (Drug-eluting Balloon in ST-Segment Elevation Myocardial Infarction) Trial. Cathet. Cardiovasc. Interv. 86, S34–S44. 10.1002/ccd.26060 26119971

[B30] Schapiro-Dufour E. Tricotel A. Slama M. S. Ducimetière P. Trinh-Duc A. Sichel C. (2019). Major Ischaemic and Bleeding Risks Following Current Drug-Eluting Stent Implantation: Are There Differences across Current Drug-Eluting Stent Types in Real Life? Archives Cardiovasc. Dis. 112, 469–484. 10.1016/j.acvd.2019.04.007 31262635

[B31] Shin E.-S. Ann S. H. Balbir Singh G. Lim K. H. Kleber F. X. Koo B.-K. (2016). Fractional Flow Reserve-Guided Paclitaxel-Coated Balloon Treatment for De Novo Coronary Lesions. Cathet. Cardiovasc. Interv. 88, 193–200. 10.1002/ccd.26257 26423017

[B32] Speck U. Cremers B. Kelsch B. Biedermann M. Clever Y. P. Schaffner S. (2012). Do pharmacokinetics Explain Persistent Restenosis Inhibition by a Single Dose of Paclitaxel? Circ. Cardiovasc. Interv. 5, 392–400. 10.1161/circinterventions.111.967794 22619258

[B33] Steigen T. K. Maeng M. Wiseth R. Erglis A. Kumsars I. Narbute I. (2006). Randomized Study on Simple versus Complex Stenting of Coronary Artery Bifurcation Lesions. Circulation 114, 1955–1961. 10.1161/CIRCULATIONAHA.106.664920 17060387

[B34] Vogt F. Stein A. Rettemeier G. Krott N. Hoffmann R. vom Dahl J. (2004). Long-term Assessment of a Novel Biodegradable Paclitaxel-Eluting Coronary Polylactide Stent. Eur. Heart J. 25, 1330–1340. 10.1016/j.ehj.2004.06.010 15288161

[B35] Vos N. S. Dirksen M. T. Vink M. A. van Nooijen F. C. Amoroso G. Herrman J.-P. R. (2014). Safety and Feasibility of a Paclitaxel-Eluting Balloon Angioplasty in Primary Percutaneous Coronary Intervention in Amsterdam (PAPPA): One-Year Clinical Outcome of a Pilot Study. EuroIntervention 10, 584–590. 10.4244/eijv10i5a101 25256200

[B36] Vos N. S. Fagel N. D. Amoroso G. Herrman J.-P. R. Patterson M. S. Piers L. H. (2019). Paclitaxel-Coated Balloon Angioplasty versus Drug-Eluting Stent in Acute Myocardial Infarction. JACC Cardiovasc. Interv. 12, 1691–1699. 10.1016/j.jcin.2019.04.016 31126887

[B37] Wöhrle J. Werner G. S. (2013). Paclitaxel-coated Balloon with Bare-Metal Stenting in Patients with Chronic Total Occlusions in Native Coronary Arteries. Cathet. Cardiovasc. Interv. 81, 793–799. 10.1002/ccd.24409 22511572

