# Supplementary material for: Computational Pressure-Fluid Dynamics Applied to Index of Microcirculatory Resistance, Predicting the Prognosis of Drug-Coated Balloons Compared With Drug-Eluting Stents in STEMI Patients
Source: Front Physiol. 2022 May 24;13:898659. doi: 10.3389/fphys.2022.898659 (PMC9171027; doi:10.3389/fphys.2022.898659)
Supplement: Supplementary file 1 [file DataSheet1.docx]

**Supplementary Materials**

Online **Table S1**. Baseline characteristics of the study population after propensity score matching.

Online **Table S2**. Characteristics of PPCI process after propensity score matching.

Online **Table S3**. Baseline characteristics of the study population re-grouped by MACEs after propensity score matching.

Online **Table S4**. Characteristics of PPCI process re-grouped by MACEs after propensity score matching.

Online **Table S5**. Baseline characteristics of the study population grouped by caIMR.

Online **Table S6**. Characteristics of PPCI process of the study population grouped by caIMR.

**Supplementary Tables**

| **Table S1**. Baseline characteristics of the study population after propensity score matching | | | | |
| --- | --- | --- | --- | --- |
| Variable | Group DES | Group DCB | *p* value | Total |
| **General characteristics** | N=62 | N=62 |  | N=124 |
| Age, y, Mean (SD) | 58.45 (12.31) | 58.24 (14.50) | 0.93 | 58.35 (13.39) |
| Males, n (%) | 51 (82.26) | 54 (87.10) | 0.62 | 105 (84.68) |
| Systolic BP, mmHg, Mean (SD) | 129.92 (18.90) | 128.48 (17.53) | 0.66 | 129.20 (18.17) |
| Heart rate, /min, Mean (SD) | 79.71 (17.89) | 75.65 (11.64) | 0.14 | 77.68 (15.17) |
| Body mass index, kg/m^2^, Mean (SD) | 25.30 (5.93) | 25.07 (3.26) | 0.78 | 25.18 (4.77) |
| Hypertension, n (%) | 27 (43.55) | 28 (45.16) | 0.99 | 55 (44.35) |
| Diabetes, n (%) | 13 (20.97) | 14 (22.58) | 0.99 | 27 (21.77) |
| Smoking, n (%) | 26 (41.94) | 29 (46.77) | 0.72 | 55 (44.35) |
| Time from symptom to balloon, hours, Mean (SD) | 7.36 (3.62) | 7.08 (3.15) | 0.65 | 7.22 (3.38) |
| **Killip level, n (%)** |  |  |  |  |
| I | 56 (90.32) | 58 (93.55) | 0.34 | 114 (91.94) |
| II | 1 (1.61) | 2 (3.23) |  | 3 (2.43) |
| III | 2 (3.23) | 2 (3.23) |  | 4 (3.23) |
| IV | 3 (4.84) | 0 |  | 3 (2.42) |
| **Baseline LVEF and biomarkers** |  |  |  |  |
| LVEF, %, Mean (SD) | 46.63 (10.48) | 56.81 (6.93) | **<0.01** | 51.72 (10.21) |
| Peak hsTnT, ng/L, Median (IQR) | 3959.00 (4331.75) | 1872.50 (8411.83) | 0.09 | 4062.72 (3441.09) |
| Peak CK-MB, ng/L, Median (IQR) | 136.20 (201.63) | 74.33 (95.08) | 0.09 | 137.89 (93.37) |
| CRP, Mean (SD) | 13.05 (10.76) | 10.84 (9.48) | 0.23 | 11.95 (10.16) |
| Serum Creatinine, umol/L, Mean (SD) | 67.89 (15.44) | 68.03 (20.36) | 0.96 | 67.96 (17.99) |
| LDL-C, mmol/L, Mean (SD) | 2.78 (1.14) | 2.60 (0.79) | 0.31 | 2.69 (0.98) |
| **Medication, n (%)** |  |  |  |  |
| Asprin | 62 (100) | 62 (100) | NA | 124 (100) |
| P2Y12 inhibitors | 62 (100) | 62 (100) | NA | 124 (100) |
| Statins | 61 (98.39) | 59 (95.16) | 0.62 | 120 (96.77) |
| Beta-blocker | 56 (90.32) | 50 (80.65) | 0.20 | 106 (85.48) |
| RAASI | 46 (74.19) | 39 (62.90) | 0.25 | 85 (68.5) |
| IV diuretics | 30 (48.39) | 12 (19.35) | **<0.01** | 42 (33.87) |

Abbreviations: DES, drug-eluting stents; DCB, drug-coated balloons; SD, standard deviation; IQR, inter-quartile range; BP, blood pressure;

PCI, percutaneous coronary intervention; AMI, acute myocardial infarction; LVEF, left ventricular ejection fraction; hsTnT, high sensitivity

troponin T; CK-MB, MB isoenzyme of creatine kinase; CRP, C-reactive protein; LDL-C, low-density lipoprotein cholesterol; RAASI,

renin-angiotensin-aldosterone system inhibitor; IV diuretics, Intravenous diuretics; NA, not available.

| **Table S2**. Characteristics of PPCI process of the study population after propensity score matching | | | | |
| --- | --- | --- | --- | --- |
| Variable | Group DES | Group DCB | *p* value | Total |
|  | N=62 | N=62 |  | N=124 |
| caIMR, Mean (SD)* | 38.68 (22.66) | 35.86 (24.89) | 0.52 | 37.25 (23.76) |
| caIMR>40, n (%) * | 22 (37.93) | 19 (31.67) | 0.48 | 41 (34.75) |
| **Criminal vessel, n (%)** |  |  |  |  |
| Left anterior descending | 33 (53.20) | 24 (38.70) | >0.05 | 57 (46.00) |
| Left circumflex | 7 (11.29) | 19 (30.60) |  | 26 (21.00) |
| Right coronary artery | 22 (35.48) | 19 (30.60) |  | 41 (33.06) |
| Multi coronary artery lesions, n (%) | 41 (66.13) | 35 (56.45) | 0.36 | 76 (61.29) |
| IABP, n (%) | 1 (1.61) | 0 | 0.99 | 1 (0.81) |
| Pre-dilated balloon diameter, mm, Mean (SD) | 2.14 (0.27) | 2.55 (0.47) | **<0.01** | 2.34 (0.43) |
| Pre-dilated balloon pressure, atm, Mean (SD) | 9.61 (2.15) | 9.74 (2.50) | 0.76 | 9.68 (2.32) |
| DES/DCB diameter, mm, Mean (SD) | 3.08 (0.47) | 2.71 (0.52) | **<0.01** | 2.89 (0.53) |
| DES/DCB length, mm, Mean (SD) | 28.05 (6.40) | 24.44 (5.34) | **<0.01** | 26.24 (6.14) |
| DES/DCB dilation released pressure, atm, Mean (SD) | 11.92 (2.32) | 9.44 (2.08) | **<0.01** | 10.68 (2.52) |
| DES/DCB dilation duration, second, Mean (SD) | 10.00 (0.15) | 69.27 (17.34) | **<0.01** | 39.64 (32.17) |
| *DES group included 62 patients; DCB groups included 62 patients.  Abbreviations: PPCI, primary percutaneous coronary intervention; caIMR, coronary-angiography-derived index of microcirculatory resistance; DES, drug-eluting stents; DCB, drug-coated balloons; SD, standard deviation; IABP, intra-aortic balloon pump; NA, not available. | | | | |

| **Table S3**. Baseline characteristics of the study population grouped by MACEs after propensity score matching | | | |
| --- | --- | --- | --- |
| Variable | N-MACE | MACE | *p* value |
| **General characteristics** | N=103 | N=21 |  |
| Age, y, Mean (SD) | 57.50 (13.10) | 62.52 (14.36) | 0.12 |
| Males, n (%) | 92 (89.32) | 13 (61.90) | **<0.01** |
| Systolic BP, mmHg, Mean (SD) | 129.19 (18.12) | 129.24 (18.84) | 0.99 |
| Heart rate, /min, Mean (SD) | 77.91 (15.36) | 76.52 (14.50) | 0.70 |
| Body mass index, kg/m^2^, Mean (SD) | 25.28 (5.02) | 24.73 (3.36) | 0.63 |
| Hypertension, n (%) | 43 (41.75) | 12 (57.14) | 0.23 |
| Diabetes, n (%) | 24 (23.30) | 3 (14.29) | 0.36 |
| Smoking, n (%) | 46 (44.66) | 9 (42.86) | 0.88 |
| Time from AMI onset to PCI, hours, Mean (SD) | 6.67 (3.31) | 9.94 (2.26) | **<0.01** |
| **Killip level, n (%)** |  |  |  |
| I | 95 (92.23) | 19 (90.48) | 0.71 |
| II | 2 (1.94) | 1 (4.76) |  |
| III | 3 (2.91) | 1 (4.76) |  |
| IV | 3 (2.91) | 0 |  |
| **Baseline LVEF and biomarkers** | |  |  |
| LVEF, %, Mean (SD) | 52.84 (10.01) | 46.19 (9.55) | **0.01** |
| Peak hsTnT, ng/L, Median (IQR) | 3306.00 (5353.00) | 2116.00 (4997.00) | 0.19 |
| Peak CK-MB, ng/L, Median (IQR) | 69.27 (121.60) | 236.48 (130.30) | **<0.01** |
| CRP, Mean (SD) | 11.58 (10.00) | 13.72 (10.98) | 0.38 |
| Serum Creatinine, umol/L, Mean (SD) | 68.36 (18.61) | 66.00 (14.86) | 0.53 |
| LDL-C, mmol/L, Mean (SD) | 2.69 (0.99) | 2.69 (0.97) | 0.99 |
| **Medication, n (%)** | |  |  |
| Asprin | 103 (100) | 21 (100) | NA |
| P2Y12 inhibitors | 103 (100) | 21 (100) | NA |
| Statins | 99 (96.12) | 21 (100) | 0.36 |
| Beta-blocker | 88 (85.44) | 18 (85.71) | 0.97 |
| RAASI | 70 (67.96) | 15 (71.43) | 0.76 |
| IV diuretics | 31 (30.10) | 11 (52.38) | 0.05 |
| Abbreviations: DES, drug-eluting stents; DCB, drug-coated balloons; SD, standard deviation; IQR, inter-quartile range; BP, blood pressure; PCI, percutaneous coronary intervention; AMI, acute myocardial infarction; LVEF, left ventricular ejection fraction; hsTnT, high sensitivity troponin T; CK-MB, MB isoenzyme of creatine kinase; CRP, C-reactive protein; LDL-C, low-density lipoprotein cholesterol; RAASI, renin-angiotensin-aldosterone system inhibitor; IV diuretics, Intravenous diuretics; NA, not available. | | | |

| **Table S4**. Characteristics of PPCI process of the study population grouped by MACEs after propensity score matching | | | |
| --- | --- | --- | --- |
| Variable | N-MACE | MACE | *p* value |
|  | N=103 | N=21 |  |
| DCB Intervention, n (%) | 56 (54.37) | 6 (28.57) | **0.03** |
| caIMR, Mean (SD)* | 36.73 (23.80) | 39.81 (23.99) | 0.60 |
| caIMR>40, n (%) * | 33 (33.67) | 8 (40.00) | 0.59 |
| Door to balloon, minutes, Mean (SD) | 62.96 (14.43) | 88.95 (4.28) | **<0.01** |
| **Criminal vessel, n (%)** |  |  |  |
| Left anterior descending | 45 (43.70) | 12 (57.10) | 0.50 |
| Left circumflex | 23 (22.30) | 3 (14.29) |  |
| Right coronary artery | 35 (34.00) | 6 (28.60) |  |
| Multi coronary artery lesions, n (%) | 65 (63.11) | 11 (52.38) | 0.36 |
| IABP, n (%) | 1 (0.97) | 0 | 0.65 |
| Pre-dilated balloon diameter, mm, Mean (SD) | 2.38 (0.45) | 2.19 (0.31) | **0.03** |
| Pre-dilated balloon pressure, atm, Mean (SD) | 9.77 (2.45) | 9.24 (1.48) | 0.20 |
| DES/DCB diameter, mm, Mean (SD) | 2.89 (0.54) | 2.90 (0.48) | 0.91 |
| DES/DCB length, mm, Mean (SD) | 25.70 (6.31) | 28.90 (4.50) | **0.01** |
| DES/DCB dilation released pressure, atm, Mean (SD) | 10.49 (2.52) | 11.62 (2.40) | 0.06 |
| DES/DCB dilation duration, second, Mean (SD) | 42.52 (32.61) | 25.48 (26.17) | **0.01** |
| *DES group included 62 patients; DCB groups included 62 patients.  Abbreviations: PPCI, primary percutaneous coronary intervention; caIMR, coronary-angiography-derived index of microcirculatory resistance; DES, drug-eluting stents; DCB, drug-coated balloons; SD, standard deviation; IABP, intra-aortic balloon pump; NA, not available. | | | |

| **Table S5**. Baseline characteristics of the study population grouped by caIMR | | | |
| --- | --- | --- | --- |
| Variable | caIMR <=40U | caIMR > 40U | *P* value |
| **General characteristics** | N=138 | N=63 |  |
| Age, y, Mean (SD) | 58.48 (13.44) | 58.75 (12.62) | 0.89 |
| Males, n (%) | 114 (82.60) | 54 (85.70) | 0.68 |
| Systolic BP, mmHg, Mean (SD) | 125.96 (19.26) | 127.52 (16.55) | 0.58 |
| Heart rate, /min, Mean (SD) | 77.12 (14.05) | 79.41 (17.26) | 0.32 |
| Body mass index, kg/m^2^, Mean (SD) | 25.35 (3.63) | 25.97 (5.43) | 0.34 |
| Hypertension, n (%) | 69 (50.00) | 32 (50.80) | 0.99 |
| Diabetes, n (%) | 26 (18.80) | 9 (14.30) | 0.55 |
| Smoking, n (%) | 73 (52.90) | 24 (38.10) | 0.07 |
| Time from symptom to balloon, hours, Mean (SD) | 6.95 (3.23) | 6.68 (3.35) | 0.59 |
| **Killip level, n (%)** |  |  |  |
| I | 126 (91.30) | 57 (90.50) | 0.81 |
| II | 6 (4.30) | 5 (7.90) |  |
| III | 4 (2.90) | 0 |  |
| IV | 2 (1.40) | 1 (1.60) |  |
| **Baseline LVEF and biomarkers** |  |  |  |
| LVEF, %, Mean (SD) | 52.40 (9.78) | 51.56 (9.82) | 0.57 |
| Peak hsTnT, ng/L, Median (IQR) | 2373.50 (4965.97) | 3114.00 (4255.00) | 0.92 |
| Peak CK-MB, ng/L, Median (IQR) | 93.37 (125.47) | 86.54 (161.87) | 0.55 |
| CRP, Mean (SD) | 11.05 (9.62) | 13.70 (11.48) | 0.09 |
| Serum Creatinine, umol/L, Mean (SD) | 68.20 (16.63) | 68.13 (15.80) | 0.98 |
| LDL-C, mmol/L, Mean (SD) | 2.64 (0.90) | 2.64 (0.89) | 0.96 |
| **Medication, n (%)** |  |  |  |
| Asprin | 138 (100) | 63 (100) | NA |
| P2Y12 inhibitors | 138 (100) | 63 (100) | NA |
| Statins | 136 (98.60) | 60 (95.20) | 0.18 |
| Beta-blocker | 118 (85.50) | 56 (88.90) | 0.66 |
| RAASI | 96 (69.60) | 45 (71.40) | 0.87 |
| IV diuretics | 51 (37.00) | 26 (41.30) | 0.64 |
| Abbreviations: HF, heart failure; SD, standard deviation; IQR, inter-quartile range; BP, blood pressure; PCI, percutaneous coronary intervention; AMI, acute myocardial infarction; LVEF, left ventricular ejection fraction; hsTnT, high sensitivity troponin T; CK-MB, MB isoenzyme of creatine kinase; CRP, C-reactive protein; LDL-C, low-density lipoprotein cholesterol; RAASI, renin-angiotensin-aldosterone system inhibitor; IV diuretics, Intravenous diuretics; NA, not available. | | | |

| **Table S6**. Characteristics of PPCI process of the study population grouped by caIMR | | | |  |
| --- | --- | --- | --- | --- |
| Variable | caIMR <=40U | caIMR > 40U | *P* |  |
|  | N=138 | N=63 | value |  |
| caIMR, Mean (SD) | 22.85 (8.43) | 63.46 (16.83) | **<0.01** |  |
| Time from Door to balloon, minutes, Mean (SD) | 66.57 (15.98) | 69.16 (14.98) | 0.28 |  |
| DCB Intervention, n (%) | 59 (42.80) | 22 (34.90) | 0.35 |  |
| **Criminal vessel, n (%)** |  |  |  |  |
| Left anterior descending | 52 (37.70) | 34 (54.00) | **0.03** |  |
| Left circumflex | 23 (16.70) | 13 (20.60) | 0.55 |  |
| Right coronary artery | 63 (45.70) | 16 (25.40) | **0.01** |  |
| Multi coronary artery lesions, n (%) | 90 (65.20) | 34 (54.00) | 0.16 |  |
| IABP, n (%) | 2 (1.40) | 1 (1.60) | 1.00 |  |
| Pre-dilated balloon diameter, mm, Mean (SD) | 2.36 (0.47) | 2.33 (0.52) | 0.75 |  |
| Pre-dilated balloon pressure, atm, Mean (SD) | 9.78 (2.13) | 9.71 (2.24) | 0.84 |  |
| DES/DCB diameter, mm, Mean (SD) | 2.94 (0.52) | 2.96 (0.50) | 0.78 |  |
| DES/DCB length, mm, Mean (SD) | 26.51 (6.73) | 26.62 (5.52) | 0.91 |  |
| DES/DCB dilation released pressure, atm, Mean (SD) | 10.76 (2.58) | 11.10 (2.78) | 0.41 |  |
| DES/DCB dilation duration, second, Mean (SD) | 35.83 (31.94) | 29.84 (28.20) | 0.18 |  |
| Abbreviations: PPCI, primary percutaneous coronary intervention; caIMR, coronary-angiography-derived index of microcirculatory resistance; DES, drug-eluting  stents; DCB, drug-coated balloons; SD, standard deviation; IABP, intra-aortic balloon pump; NA, not available. | | | | |
